# Technological Resources for Physical Rehabilitation in Cancer Patients Undergoing Chemotherapy: A Scoping Review

**DOI:** 10.3390/cancers16233949

**Published:** 2024-11-25

**Authors:** Anabela Amarelo, Marisa Mota, Bruno Amarelo, Marta Campos Ferreira, Carla Sílvia Fernandes

**Affiliations:** 1 Gaia-Espinho Local Health Unit, 4434-502 Vila Nova de Gaia, Portugal; anabela.amarelo@ulsge.min-saude.pt (A.A.); marisa.mota@ulsge.min-saude.pt (M.M.); bruno.amarelo@ulsge.min-saude.pt (B.A.); 2 Association for Research and Supportive Care in Oncology (AICSO), 4410-406 Arcozelo, Portugal; 3 INESC TEC—Institute for Systems and Computer Engineering, Technology and Science, Faculty of Engineering, University of Porto (FEUP), 4200-465 Porto, Portugal; mferreira@fe.up.pt; 4 Nursing School of Porto, 4200-072 Porto, Portugal; 5 Research Center RISEHealth, 4200-319 Porto, Portugal; 6 Association ADITGames, 4490-582 Póvoa de Varzim, Portugal

**Keywords:** oncology rehabilitation, digital health tools, supportive care in cancer, mHealth applications

## Abstract

**Background/Objectives**: Cancer patients undergoing chemotherapy often face challenges that reduce their physical function and quality of life. Technological resources offer innovative solutions for physical rehabilitation, but the extent of their application in this context remains unclear. This scoping review aims to explore and map the various technological tools used to support physical rehabilitation in cancer patients during chemotherapy, focusing on their potential to improve outcomes and enhance patient care. **Methods**: A scoping review was conducted following the Joanna Briggs Institute (JBI) guidelines and the PRISMA-ScR framework. Comprehensive searches were performed in the MEDLINE, CINAHL, Scopus, SPORTDiscus, and COCHRANE databases. The included studies focused on the technological resources used in physical rehabilitation for cancer patients undergoing chemotherapy. Data extraction followed the World Health Organization’s “Classification of Digital Health Interventions v1.0” to categorize the technologies. **Results**: A total of 32 studies met the inclusion criteria. The most commonly used technologies included wearable devices (16 studies), web-based platforms and telerehabilitation systems (7 studies), mHealth applications (6 studies), virtual reality (2 studies), and exergaming (3 studies). These tools were designed to enhance physical function, manage treatment-related symptoms, and improve overall quality of life. Wearable devices were particularly effective for monitoring physical activity, while web-based platforms and mHealth applications supported remote rehabilitation and patient engagement. **Conclusions**: Technological resources offer significant opportunities for personalized rehabilitation interventions in cancer patients undergoing chemotherapy. However, further research is needed to evaluate the long-term effectiveness, cost-efficiency, and clinical integration of these tools to ensure broader accessibility and sustainable impact.

## Simple Summary

This scoping review explores how technological tools can support cancer rehabilitation for cancer patients undergoing chemotherapy. Technologies such as wearable devices, mobile health (mHealth) applications, telerehabilitation platforms, and virtual reality have emerged as valuable aids for maintaining or improving patients’ physical activity levels. By mapping these tools, we highlight their benefits in managing cancer- and treatment-related side effects, such as fatigue and reduced mobility, ultimately enhancing quality of life. This review also identifies areas for integrating these resources into care practices and outlines directions for future research to maximize clinical effectiveness and accessibility.

## 1. Introduction

Cancer continues to be a leading public health challenge worldwide, with an estimated 20 million new cases and 10 million deaths reported in 2022 alone, according to GLOBOCAN [1,2,3]. By 2040, global cancer incidence is expected to rise to 29.9 million cases annually, with over 70 million people living after cancer, which will place an increasing burden on healthcare systems and affect the long-term well-being of survivors [3,4,5]. As the number of cancer survivors grows, the long-term effects of both the disease and its treatments become more critical to address.

Chemotherapy, despite advances in cancer treatment, remains a common therapeutic approach, either used alone or in combination with targeted and immunotherapeutic agents [6,7]. Unfortunately, chemotherapy exposes patients to a range of adverse effects, increasing morbidity and treatment costs while negatively impacting health-related quality of life (HR-QOL), and may limit the administration of future treatments, potentially affecting long-term outcomes [6,7,8].

Comprehensive care is critical for cancer patients during treatment, as prolonged periods of inactivity and the disease itself can result in fatigue, peripheral neuropathy, nausea, cardiotoxicity, cognitive impairments, and overall functional decline [7,9]. These effects often persist long after treatment, causing long-term functional challenges in returning to normal life, work, or independent living [10]. Although supportive care interventions address some of these effects, many remain difficult to manage, emphasizing the need for continuous improvements in mitigating chemotherapy-related side effects [7,8].

In these context, physical rehabilitation plays a crucial role in helping patients maintain or regain functionality during cancer treatment [10,11]. Exercise has shown substantial benefits, including improved mental health, reduced fatigue, and enhanced well-being, with guidelines recommending at least 150 min of aerobic activity and 2–3 resistance sessions per week during and after treatment [12].

Despite these benefits, several factors have been identified as barriers to exercise, including cancer therapy-related side effects, kinesiophobia, lack of access to tailored facilities, and individual health beliefs and preferences [13]. In recent years, digital technological advancements have introduced new possibilities for health [14,15,16]. As digital technologies advance, becoming smarter and interconnected through wireless communication, unimaginable possibilities open for the advancement of science, health, and rehabilitation [15].

In this setting, technologies such as mobile health applications, wearable devices, telerehabilitation platforms, and virtual reality systems are designed to remotely monitor, guide, and support physical activity and symptom management [17,18,19]. These tools enable patients and healthcare providers to engage in rehabilitation programs more efficiently, offering personalized feedback, progress tracking, and symptom management, often in real time [15,16]. However, despite the growing interest in digital health interventions within oncology, the scope of available technological resources for physical rehabilitation in cancer patients undergoing chemotherapy remains unexplored [18,20].

While existing studies suggest that mobile health applications and tele-rehabilitation tools can enhance self-management and symptom control in cancer patients, a comprehensive map of these technologies is necessary to identify trends and gaps in the literature [18,21]. This scoping review aims to map the available technological resources for physical rehabilitation in cancer patients undergoing chemotherapy, highlighting areas where further research is needed to optimize the use of technology in cancer care.

## 2. Materials and Methods

A scoping review was carried out to explore the current body of scientific literature, following the guidelines established by the Joanna Briggs Institute (JBI) [22,23]. The PRISMA-ScR framework (Preferred Reporting Items for Systematic Reviews and Meta-Analyses for Scoping Reviews) was utilized to ensure systematic organization and transparency throughout the research process, as outlined in the PRISMA-ScR guidelines [22,24,25]. The review protocol was also registered on the Open Science Framework^®^ platform, with the following https://doi.org/10.17605/OSF.IO/RV795.

### 2.1. Research Method

The research question was developed using the Population, Concept, and Context (PCC) framework, as outlined by the Joanna Briggs Institute [23,25]. The study focused on adults over the age of 18 who were cancer patients undergoing chemotherapy (Population), participating in physical rehabilitation programs (Context), and using technological resources (Concept). These criteria guided the selection of studies for the bibliographic sample, with search terms derived from this framework. To ensure broad coverage, the search included both free terms and database-specific descriptors. The search strategy was tailored for each database using English-language descriptors and syntax customized to the following platforms: MEDLINE^®^, CINAHL^®^, Scopus, SPORTDiscuss, and COCHRANE. A combination of medical subject headings (MeSHs), subject headings, and free-text terms was employed, enhancing the search’s comprehensiveness (Appendix A Table A1). Additional articles were manually added by examining the reference lists of all publications included in the review, a method referred to as “backward citation searching”. The literature search was carried out to include studies published up to 31 March 2024, with no time restrictions.

### 2.2. Eligibility Criteria

The inclusion and exclusion criteria were established based on the PCC framework, focusing on the guiding question: What technological resources are available for physical rehabilitation in cancer patients undergoing chemotherapy? Sub-questions were posed to refine the focus, exploring which types of technologies are used, what functionalities they provide, and their feasibility or acceptability.

Studies were excluded if they focused on: individuals under the age of 18; patients receiving treatments other than chemotherapy; or programs focused solely on well-being, psychological support, behavior change, or symptom management instead of physical rehabilitation. Additionally, studies focused on the prehabilitation phase or cancer survivors post-treatment were excluded, as well as non-primary research (editorials, letters, reviews, gray literature, dissertations, and book chapters). Only primary research published in English, French, Spanish, or Portuguese was considered. Qualitative, quantitative, and mixed-methods studies were included.

### 2.3. Data Extraction

The search results from each database were imported into Rayyan^®^ software (https://www.rayyan.ai, accessed on 14 August 2024), a web-based tool designed to assist in the systematic review process, to streamline the review process [26]. After removing duplicate references, two researchers (A.A. and C.S.F.) independently carried out the initial screening of titles and abstracts, applying the previously established inclusion and exclusion criteria. The full texts of the selected references were then obtained to finalize decisions on their inclusion [27]. Any disagreements regarding inclusion or exclusion were resolved by consulting a third researcher (B.A.) to reach a consensus.

Each selected article was assigned an identifier (S) based on the order of review. The PRISMA 2020 framework was used to organize the data and to ensure transparency and consistency throughout the screening and selection process [28].

### 2.4. Data Analysis

Data were collected using a customized extraction form, capturing the following details for each study: author, year of publication, and country; study design; study objective; participant characteristics; type of technology used; purpose; type of intervention; main instruments; and outcomes (Table 1). Data extraction and synthesis were performed independently by two researchers (A.A. and C.S.F.), with a third researcher (B.A.) available to resolve any disagreements.

To provide a structured categorization of technological resources in healthcare, data were analyzed based on the “Classification of Digital Health Interventions v1.0” from the World Health Organization [29]. This framework provides a structured approach for describing digital technologies in healthcare, categorizing them into four key target groups: clients, healthcare providers, health system managers, and data services. For this analysis, we extracted information regarding the target group and type of technological resource used in each study, aligning it with the WHO classification.

A multi-input table was created to present the relationships between the types of technologies and their targets, functionalities, and primary objectives. Table 2 offers a comprehensive overview of technological interventions. 

**Table 1:** Characteristics of studies included in the scoping review.

ID	Athor/Year/ Country	Study Design	Objective	Population	Technology	Purpose of Technological Resource	Intervention	Instruments	Outcomes
S01 [30]	Albrecht T 2024 (USA)	Single-arm pre–post-interventional study	Evaluate a web-based exercise tool for cancer patients undergoing chemotherapy	12 breast and prostate cancer patients	Web-based app	To promote exercise and monitor anxiety, depression, fatigue	12-week physical activity program	PROMIS, 6MWT	Improved anxiety, depression, walking distance
S02 [31]	Cheong I 2018 (Republic of Korea)	Prospective interventional study	Evaluate the effect of a mobile healthcare program on fatigue and strength	102 colorectal cancer patients	mHealth app + Wearable	To enhance strength and cardiorespiratory endurance	12-week rehabilitation	2MWT, IPAQ, PG-SGA, QLQ-C30	Improved strength, fatigue, endurance
S03 [32]	Qi Y 2024 (China)	Single-center, single-arm, prospective phase I study	Assess feasibility of VR and mHealth rehabilitation	123 lung, gastric, colorectal cancer patients	mHealth + VR	To improve physical and psychological health	8-week mHealth and VR rehabilitation	6MWT, PG-SGA, HADS, QLQ-C30	Improved BMI, anxiety, depression, muscle mass
S04 [33]	Wolff J 2023 (Germany)	Randomized controlled trial (RCT), with a waiting-list control	Evaluate the impact of a cancer exercise program using the PINK! app	60 breast cancer patients	PINK! app	To reduce fatigue and support mental well-being	12-week app-based coaching program	PHQ-9, QLQ-C30, IPAQ	Reduced fatigue, psychological distress
S05 [34]	Feyzioğlu Ö 2022 (Turkey)	Pilot study with a pre-test and post-test design	Evaluate the effectiveness of video game-based exercises for upper extremity function	30 breast cancer patients	Video game-based exercises	To improve upper extremity functionality through interactive gaming	Kinect-based exergaming	DASH, SPADI, ROM	Improved upper extremity functionality
S06 [35]	Ariza-Garcia A 2019 (Spain)	Two-arm, assessor-blinded, parallel, randomized controlled trial	Evaluate the impact of web-based exercise program on functional capacity and strength	78 breast cancer patients (stages I-IIIA)	Web-based system	To enhance functional capacity and muscle strength	Web-based exercise program	SF-36, 6MWT	Improved functional capacity, muscle strength
S07 [36]	Troschel F 2019 (Germany)	Case report	Track physical fitness and exercise adherence in a glioblastoma patient using a sports watch	1 glioblastoma patient	Sports watch	To track fitness levels and motivate exercise	Personalized fitness program	Garmin Fitness Metrics	Improved fitness, completed marathon
S08 [37]	Coats V 2019 (Canada)	Pilot study, feasibility study	Assess feasibility and effectiveness of telerehabilitation in lung cancer patients	35 lung cancer patients	Telerehabilitation	To deliver remote rehabilitation and improve functional capacity	Supervised home-based rehabilitation	6MWT, IPAQ, FFI	Improved functional capacity, high satisfaction
S09 [38]	Van Blarigan E 2022 (USA)	Pilot RCT	Evaluate physical activity improvement through personalized walking program	45 colorectal cancer patients	Fitbit + SMS	To monitor physical activity and improve adherence	Personalized walking program	Fitbit, IPAQ	Improved physical activity, high adherence
S10 [39]	Moffet H 2015 (Canada)	Longitudinal pilot study	Evaluate telerehabilitation program for lung cancer patients	12 lung cancer patients	Telerehabilitation	To provide home-based rehabilitation with professional supervision	Home-based telerehabilitation	6MWT, CRF, IPAQ	High adherence, reliable platform
S11 [40]	Purdy G M 2022 (Canada)	Feasibility study	Evaluate the use of eHealth apps for physical fitness in multiple myeloma patients	15 multiple myeloma patients	eHealth app	To enhance physical fitness and quality of life	eHealth-based exercise program	QLQ-C30, SF-36, BFI	Improved fitness, quality of life
S12 [41]	Wolff J 2024 (Germany)	Retrospective observational study (real-world data)	Assess the impact of a weight loss program using the PINK! Coach app in breast cancer survivors	100 breast cancer survivors	PINK! Coach app	To support weight loss and physical activity remotely	App-based weight loss program	BMI, 6MWT, IPAQ	Reduced BMI, increased physical activity
S13 [42]	Poh Loh K 2021 (USA)	Qualitative study	Evaluate the feasibility of a walking and resistance exercise program in myeloid neoplasm patients	20 myeloid neoplasm patients	GO-EXCAP mobile app	To facilitate walking and resistance exercise programs remotely	Walking + resistance exercise	IPAQ, SF-36	Feasible exercise program
S14 [43]	Romero-Elías M 2024 (Spain)	Qualitative study, quasi-experimental research	Evaluate psychological support and physical activity engagement through video conferencing in colorectal cancer patients	22 colorectal cancer patients	Video conferencing	To offer remote psychological support and engage in physical activity	Remote exercise program + mental health	IPAQ, SF-36	Improved psychological well-being, PA
S15 [44]	Nyrop 2017 (USA)	Longitudinal, observational study	Evaluate the impact of Fitbit monitoring on walking adherence in breast cancer patients	56 breast cancer patients	Fitbit Zip	To monitor steps and adherence to exercise	Walking program with step monitoring	Fitbit, IPAQ	Improved walking adherence, reduced fatigue
S16 [45]	Park 2019 (Republic of Korea)	Prospective, single-arm intervention study (pilot study)	Evaluate exercise capacity and distress management through smartphone app	43 NSCLC patients	Smartphone app	To track exercise capacity and provide distress management	Exercise program with remote monitoring	6MWT, PG-SGA, HADS	Improved exercise capacity, reduced distress
S17 [46]	Villaron 2018 (France)	Randomized pilot study	Assess step-tracking and telehealth for fatigue reduction and physical activity promotion	75 cancer patients (various types)	Pedometer, Telehealth	To promote physical activity and reduce fatigue through step tracking	Walking-based physical activity	IPAQ, SF-36	Improved physical activity, reduced fatigue
S18 [47]	Backman 2014 (Sweden)	Randomized controlled trial (RCT)	Evaluate pedometer-based physical activity interventions for cancer patients	97 breast and colorectal cancer patients	Pedometer	To encourage physical activity and track steps	Walking program with pedometer	IPAQ, QLQ-C30	Improved physical activity, breast cancer symptoms
S19 [48]	Gokal 2015 (UK)	Randomized controlled trial (RCT)	Evaluate the impact of a walking program on fatigue and physical activity in breast cancer patients	60 breast cancer patients	Pedometer	To promote walking and reduce fatigue	Walking program with step goals	PFS, FACIT-F, IPAQ	Improved physical activity, fatigue, self-esteem
S20 [49]	Jarden 2016 (Denmark)	Randomized controlled trial (RCT)	Evaluate the effect of multimodal rehabilitation on quality of life in acute leukemia patients	102 acute leukemia patients	Pedometer, Multimodal intervention	To track physical activity and improve quality of life	Multimodal rehab with walking program	SF-36, QLQ-C30, IPAQ	Improved physical function, quality of life
S21 [50]	Delrieu 2020 (France)	Single-arm intervention study (feasibility study)	Assess the feasibility of activity trackers in metastatic breast cancer patients	40 metastatic breast cancer patients	Activity tracker	To monitor and increase physical activity	Activity tracker-based PA program	6MWT, IPAQ	Increased physical activity, reduced sitting time
S22 [51]	Edbrooke 2019 (Australia)	Randomized controlled trial (RCT)	Evaluate SenseWear accelerometer for tracking physical activity and intensity in lung cancer patients	56 lung cancer patients	SenseWear accelerometer	To track physical activity and provide feedback on intensity	SenseWear-based activity program	6MWT, SF-36, IPAQ	Improved physical activity, HRQoL
S23 [52]	Gandhi 2020 (India)	Non-randomized controlled trial	Assess the impact of a walking program with pedometer for fatigue and QoL in breast cancer patients	85 breast cancer patients	Pedometer	To track steps and monitor fatigue	Walking program with pedometer	FACIT-F, SF-36, IPAQ	Reduced fatigue, improved quality of life
S24 [53]	Lowe 2013 (Canada)	Quasi-experimental pilot study (case series)	Evaluate the impact of an accelerometer for tracking sedentary behavior in advanced cancer patients	30 advanced cancer patients	activPAL accelerometer	To track sedentary behavior and physical activity	Sedentary behavior intervention	activPAL metrics	Improved quality of life, mixed physical function
S25 [54]	Marthick 2018 (Australia)	Prospective cohort study	Assess daily physical activity tracking in various cancer patients using Misfit Shine	112 cancer patients	Misfit Shine	To monitor daily physical activity levels	Physical activity intervention	Misfit Shine metrics	Improved physical activity, quality of life
S26 [55]	Nilsson 2020 (Norway)	Prospective cohort study	Evaluate SenseWear Armband for moderate-to-vigorous physical activity tracking in cancer patients	90 cancer patients	SenseWear Armband	To track moderate-to-vigorous physical activity	SenseWear-based PA program	MVPA metrics, SF-36	Reduced fatigue, increased MVPA
S27 [56]	Vallance 2016 (Canada)	Randomized controlled trial (RCT)	Evaluate pedometer-based walking program for physical activity promotion in breast cancer patients	81 breast cancer patients	Pedometer, print materials	To encourage adherence to a physical activity program	Walking program with pedometer	SF-36, IPAQ	High adherence, no significant difference in PA
S28 [57]	Mouri 2018 (Japan)	Prospective, multicenter, single-arm study	Evaluate walking-based outdoor activity program for physical activity in NSCLC and pancreatic cancer patients	59 NSCLC and pancreatic cancer patients	Pedometer	To promote outdoor activity and improve quality of life	Walking-based PA program	6MWT, IPAQ	Increased outdoor activity, improved QoL
S29 [58]	Parker 2019 (USA)	Prospective single-arm study	Evaluate ActiGraph accelerometer for monitoring activity in pancreatic cancer patients	47 pancreatic cancer patients	ActiGraph accelerometer	To monitor activity levels and increase moderate-to-vigorous physical activity	Personalized physical activity intervention	ActiGraph, MVPA metrics	High adherence, increased MVPA
S30 [59]	de Oliveira 2018 (Brazil)	Controlled trial	Evaluate Kinect-based rehabilitation for fatigue reduction in cancer patients	60 cancer patients	Xbox Kinect	To promote interactive rehabilitation and reduce fatigue	Kinect-based rehabilitation program	FACIT-F, SF-36	Reduced fatigue, improved quality of life
S31 [60]	de Oliveira 2020 (Brazil)	Randomized controlled clinical trial	Assess the impact of Kinect-based exercises on shoulder mobility and disability	50 cancer patients	Xbox Kinect	To improve shoulder mobility and reduce disability	Kinect-based exercise program	DASH, ROM	Reduced shoulder disability, no change in muscle strength
S32 [61]	Tsuda 2016 (Japan)	Prospective single-arm feasibility study	Evaluate the use of Nintendo Wii Fit for maintaining physical performance in hematologic malignancy patients	22 hematologic malignancy patients	Nintendo Wii Fit	To maintain physical performance and improve psychological health	Wii Fit-based exercise program	HADS, PG-SGA, 6MWT	Maintained physical performance, improved anxiety and depression

6MWT: 6-Minute Walk Test; 2MWT: 2-Minute Walk Test; BFI: Brief Fatigue Inventory; BMI: Body Mass Index; CRF: cancer-related fatigue; DASH: disabilities of the arm, shoulder, and hand; FACIT-F: Functional Assessment of Chronic Illness Therapy—Fatigue; FFI: Foot Function Index; HADS: Hospital Anxiety and Depression Scale; IPAQ: International Physical Activity Questionnaire; MVPA: moderate-to-vigorous physical activity; PG-SGA: Patient-Generated Subjective Global Assessment; PHQ-9: Patient Health Questionnaire-9; PROMIS: Patient-Reported Outcomes Measurement Information System; PFS: Piper Fatigue Scale; QLQ-C30: Quality of Life Questionnaire—Core 30; ROM: range of motion; SF-36: Short-Form (36) Health Survey; SPADI: Shoulder Pain and Disability Index.

## 3. Results

**Figure 1:**
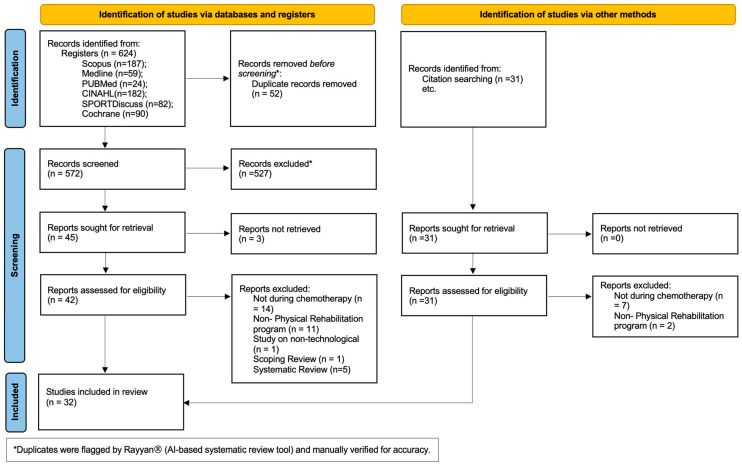
Article identification and inclusion process—PRISMA diagram flow (2020).

A total of 624 articles were retrieved from the initial search across various databases. After removing duplicates, 572 articles remained for the initial screening based on title and abstract. Forty-five articles were selected for full-text review, including those identified through backward citation searching. Ultimately, 32 studies met the inclusion criteria for analysis (Figure 1) [28].

A detailed summary of the included studies, including information on the authors, year and country of publication, study design, objective, population characteristics, technology type, objectives, interventions, measurement tools, and outcomes, is presented in Table 1.

### 3.1. Characteristics of the Included Studies

Although there was no time limit set, the included studies span from 2013 to 2024 [27,28,29,30,31,32,33,34,35,36,37,38,39,40,41,42,43,44,45,46,47,48,49,50,51,52,53,54,55,56,57,58], with the majority published between 2018 and 2024 (S01, S03, S04, S05, S08, S09, S10, S11, S12, S13, S14, S16, S21, S22, S23, S26, S27, S29, and S31). The studies were conducted in various countries, highlighting the global interest in technological interventions for cancer rehabilitation. The most represented countries are the USA (S01, S09, S12, S13, S15, S19, and S29), Canada (S08, S10, S11, S24, and S27), and Germany (S04, S07, S12, and S17), with additional studies from every part of the globe (Brazil, Republic of Korea, China, Australia, Japan, France, Denmark, Sweden, Norway, and Turkey).

The study designs varied, with randomized controlled trials (RCTs) being the most common, used in nine studies (S04, S06, S09, S18, S19, S20, S22, S27, S31). This was followed by single-arm interventional studies, featured in eight studies (S01, S03, S09, S10, S12, S23, S29, S32). Pilot studies were conducted in five studies (S05, S08, S09, S10, S17), while feasibility studies appeared in four studies (S08, S11, S21, S32). Additionally, non-randomized controlled trials were used in two studies (S23, S30), and qualitative studies were included in two studies (S13, S14). The review also included one case report (S07) and two longitudinal observational studies (S15, S24).

The studies included in this review encompassed a diverse range of cancer patients, with populations varying in size, cancer type, and treatment phase. Most studies focused on patients undergoing chemotherapy at various stages of their treatment. A significant number of studies specifically targeted patients actively undergoing chemotherapy, including S01, S02, S04, S06, S08, S10, S11, S13, S15, S17, S18, and S19. Other studies included patients receiving multiple treatments, such as chemotherapy combined with other modalities (S03, S05, S07, S09, S12, S16, S20, S21, S23, and S32). A few studies focused on other treatment phases, such as cancer survivorship (S14, S24, S25). Sample sizes varied across the studies. The smallest study involved a single patient (S07), while the largest primary study included 112 participants (S25). Common cancer types were well-represented, including breast cancer (S01, S04, S05, S06, S12, S15, S18, S19, S21, S23, S27), colorectal cancer (S02, S09, S14, S23), lung cancer (S03, S08, S10, S13, S16, S22), multiple myeloma (S11, S13), and hematologic malignancies (S20, S32).

### 3.2. Characteristics of Technological Resources

Each technology in the reviewed studies was carefully implemented to address specific rehabilitation goals, including the promotion of physical activity, improvement of mental health, reduction in fatigue, and enhancement of quality of life for cancer patients during and after chemotherapy. The wide array of technological resources used in these interventions, such as wearable devices, mHealth applications, telerehabilitation platforms, VR, and AVGs, reflects the diverse approaches to supporting cancer rehabilitation. These resources can be systematically classified using the WHO Digital Health Intervention (DHI) v1.0 framework [29], which organizes interventions based on the target group—clients (e.g., self-monitoring and coaching), healthcare providers, or health systems. The majority of the interventions targeted clients, focusing on promoting physical activity, monitoring health parameters, and managing symptoms (DHI 1.1–1.4), as outlined in Table 1 [29].

Wearable devices emerged as one of the most widely used tools, particularly for monitoring physical activity, vital signs, and sleep patterns. According to the WHO DHI classification, these devices fall under DHI 1.2: Client-targeted monitoring interventions (S02, S07, S09, S15, S17-S24, S26-S29) [26]. For example, wearables like Fitbit (S09, S15) were commonly used to track steps and physical activity, while accelerometers such as the SenseWear (S22, S26) and ActiGraph (S29) provided continuous monitoring of physical activity and physiological metrics like heart rate and step count. Additionally, sports watches like the Polar V800 (S07) played a role in tracking exercise intensity during high-intensity training programs.

In conjunction with wearables, mHealth applications (apps) were frequently deployed to offer additional functionalities such as feedback, progress monitoring, and tailored rehabilitation programs. In studies like S02, S03, S04, S12, S13, and S16, these applications facilitated physical activity tracking and often included components such as dietary guidance, symptom management, and mental health support. These mHealth apps are categorized under DHI 1.1: Client-targeted digital interventions [29]. Notable examples include the PINK! app (S04, S12), which supported physical activity and provided nutritional and mental health guidance. Similarly, apps like GO-EXCAP (S13) and other smartphone-based applications (S16) facilitated exercise capacity tracking while offering psychological support to patients.

In addition to wearables and mobile apps, digital platforms (S01, S06, S08, S10, S11, S14, S25) enabled patients to participate in home-based rehabilitation programs, with real-time supervision and monitoring of physical exercises. These platforms, classified under DHI 1.4: Client-targeted support for rehabilitation and management, allowed healthcare providers to engage with patients remotely, offering interactive components that enhanced patient engagement and adherence to their rehabilitation programs [29].

Technologies also extended beyond monitoring and feedback into immersive environments such as VR-based interventions (S03, S32), which fall under DHI 1.4 [29]. These VR-based tools combined physical therapy with psychological support to improve both physical and mental well-being during cancer treatment.

AVGs were another innovative approach used in studies such as S05, S30, S31 and S32. Through exergames like Xbox Kinect and Nintendo Wii, patients engaged in gamified exercise programs aimed at improving endurance, strength, and functionality. These interventions are categorized under DHI 1.3: Client-targeted digital health education and training [29].

Furthermore, web-based platforms like the e-CuidateChemo platform (S08) and the Exercise Planning and Tracking (EPT) tool (S01) provided structured exercise programs for patients at home, mitigating physical decline associated with chemotherapy. These systems are classified as DHI 1.1: Client-targeted digital interventions for self-management [29], emphasizing their role in empowering patients to take control of their rehabilitation.

In summary, most of the studies (*n* = 13) focused on client-targeted interventions (DHI 1.0), with the primary goal of promoting physical activity, monitoring symptoms, and managing rehabilitation remotely [29]. Table 2 provides a comprehensive overview of the technological resources used in these studies, their WHO DHI classifications, functionalities, and key findings.

### 3.3. Technological Functionalities

**Figure 2:**
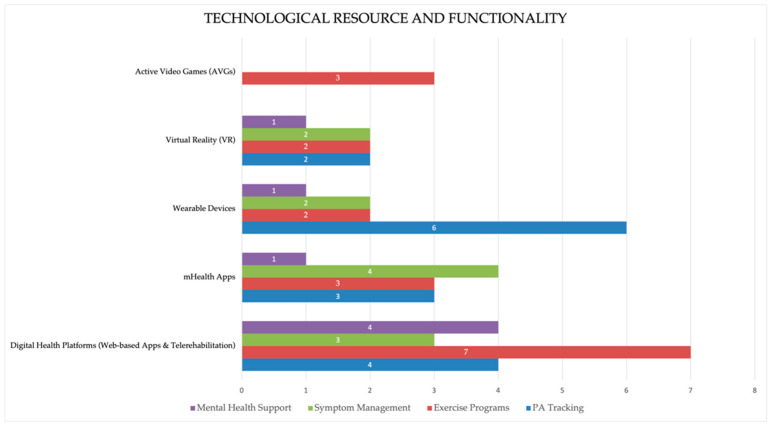
Distribution of technology types and functionalities.

The technological resources employed across the studies served a wide range of functionalities, categorized according to the WHO’s DHI v1.0 classification [29]. A graphical representation of the technological resources and their functionalities is provided in Figure 2, illustrating the distribution of technologies across different categories, including physical activity tracking, exercise programs, symptom management, and mental health support.

A primary functionality observed was “client-targeted tools for self-monitoring” of physical activity (DHI 1.2), predominantly using wearables. These devices, including Fitbits, accelerometers, and pedometers, were widely used to monitor exercise intensity, track steps, and assess physical activity levels across studies like S02, S03, S09, S11, S13, S16, S22, S23, S25, and S29. Increased adherence to physical activity guidelines was commonly reported with these tools. For instance, the use of Fitbits in S09 was associated with improved step counts and walking distance. Similarly, pedometers and activity trackers in S21, S23, and S25 were linked to enhanced physical activity levels and reductions in sedentary behavior.

Beyond monitoring, several technologies also supported structured exercise programs, classified under “client-targeted therapy” (DHI 1.1). Mobile Health applications (e.g., S02, S05) and telerehabilitation platforms (e.g., S08, S10, S13) allowed patients to engage in structured exercise regimens incorporating both strength and aerobic training. Notably, studies like S01, S06, S12, S18, S20, S30, and S31 demonstrated how web-based or interactive rehabilitation tools contributed to improvements in physical function, endurance, and muscle strength. Furthermore, exergaming technologies in studies such as S20, S30, and S31 provided engaging, interactive exercises, enhancing upper extremity functionality and shoulder mobility.

In addition to physical rehabilitation, several technological resources extended their functionalities to include symptom management and mental health support. For example, wearables in studies like S03 and S13 helped monitor symptoms such as fatigue and psychological distress, enabling patients to manage both the physical and emotional impacts of their treatment. Similarly, VR-based interventions (e.g., S03, S30) contributed not only to physical rehabilitation but also to the improvement of mental health outcomes, reducing anxiety and depression. The PINK! app (e.g., S04, S12) effectively supported mental well-being by reducing psychological distress and fatigue in breast cancer patients.

## 4. Discussion

This scoping review highlights the potential of technological tools to enhance physical rehabilitation in cancer patients undergoing chemotherapy. The 32 studies analyzed employed a variety of technologies, including wearable devices, mHealth apps, digital platforms (web-based apps and telerehabilitation), VR, and exergaming systems, with a predominant focus on client-targeted functionalities. These tools were found to address critical rehabilitation needs, such as improving adherence to physical activity protocols, enhancing functional capacity, and managing chemotherapy-related side effects.

Wearable devices, such as Fitbits and accelerometers (such as SenseWear, ActiGraph and sports watches), emerged as the most frequently used technology [31,38,44,45]. Primarily serving as self-monitoring tools (DHI 1.2), wearables tracked metrics like steps, heart rate, and sleep patterns. Real-time feedback provided by these devices was shown to improve adherence to physical activity guidelines. For instance, a study in 2022 [38] demonstrated that using Fitbit increased step count and walking distance in colorectal cancer patients during chemotherapy. Similarly, research conducted in 2019 [45] showed the effectiveness of SenseWear in tracking physical performance among advanced lung cancer patients. The feasibility and usability of wearables were consistently high across studies, with patients successfully integrating these tools into their daily lives. A 2017 study [44] found that the Fitbit Zip effectively supported real-time feedback on physical activity, helping patients meet recommended activity levels during breast cancer treatment. Patients showed strong adherence to wearable devices, finding them easy to use even during demanding chemotherapy cycles, as demonstrated in studies [31,44]. These wearables, particularly Fitbit and accelerometers, proved effective in promoting adherence to physical activity, which is essential for maintaining functionality during chemotherapy.

Similarly, mHealth applications and telerehabilitation platforms also demonstrated high usability. Platforms such as eChez-Soi and GO-EXCAP provided real-time feedback and personalized interventions, which patients found intuitive and motivating [37,42]. These tools helped sustain engagement and adherence, particularly when real-time feedback was incorporated into their functionality. However, advanced technologies like VR presented challenges, as access to devices and technological literacy posed barriers for some patients, as noted in studies [32,61].

The technologies evaluated in this review were largely suitable for cancer rehabilitation. Wearable devices were particularly effective in tracking physical activity, a critical component of rehabilitation during chemotherapy [31,44]. Telerehabilitation platforms provided a valuable solution for patients with limited mobility or those living far from treatment centers, as highlighted in studies [37,39]. These platforms enabled remote supervision and interaction with healthcare providers, allowing patients to participate in structured exercise programs from home. However, more complex technologies, such as VR and exergaming, proved more beneficial for younger or tech-savvy patients who were more familiar with gaming systems, as seen in studies [46,60].

Mobile health applications were widely used across studies to support structured exercise programs and symptom management. For instance, the PINK! app was particularly effective in reducing psychological distress and fatigue among breast cancer patients, while GO-EXCAP provided tailored, home-based exercise programs that promoted patient autonomy [41,42]. These applications, categorized as client-targeted interventions for self-management (DHI 1.1), addressed areas such as physical activity, nutrition, and mental health coaching, with high feasibility and engagement, even when patients experienced treatment-related fatigue [42].

Telerehabilitation platforms and web-based applications, as demonstrated in studies such as [36,37], enabled patients to receive remote supervision for their rehabilitation exercises. These platforms, classified under remote care interventions (DHI 1.1), allowed for real-time monitoring and provided feedback, which improved functional capacity and endurance. For example, the eChez-Soi platform [37] achieved high patient satisfaction and notable functional improvements, particularly among lung cancer patients who found it difficult to attend in-person rehabilitation sessions [39].

Virtual reality and exergaming systems were explored in four studies [32,46], offering a more interactive and engaging approach to rehabilitation. Virtual reality was shown to improve body composition and reduce fatigue, as demonstrated in one study [32], while exergaming systems (e.g., Xbox Kinect) used in [46] engaged patients in gamified exercises to enhance muscle function and increase physical activity levels. Although these technologies provided high levels of engagement, their adoption was limited by patient access and familiarity with the devices.

Across the studies, the feasibility of these technologies was consistently rated as high. Wearables like Fitbits and accelerometers demonstrated strong usability and adherence, while telerehabilitation platforms provided an accessible and flexible alternative to in-person rehabilitation. However, advanced technologies like VR faced challenges related to technological literacy and access, limiting their widespread adoption.

The findings of this scoping review align with previous studies that underscore the increasing role of digital health interventions in cancer rehabilitation. Wearable devices and mHealth applications, such as those used to monitor physical activity and manage symptoms, have demonstrated significant potential in improving patient adherence to rehabilitation protocols [17,18]. These technologies not only offer real-time feedback but also facilitate self-management, resulting in improved patient outcomes and satisfaction. However, the integration of more advanced technologies like VR poses challenges, including technological literacy and access [19]. Despite these barriers, VR has been effective in reducing symptoms such as anxiety, pain, and fatigue, emphasizing its potential in cancer rehabilitation. Furthermore, a study [62] has highlighted the importance of balancing the benefits of exercise programs with the potential risks, especially for patients undergoing systemic treatments. Addressing barriers and facilitators, as identified in another study [63], is crucial for ensuring broader adoption and success of digital health tools in oncology care.

### Challenges and Limitations

This scoping review identified several challenges when analyzing technological resources for physical rehabilitation in cancer patients undergoing chemotherapy. A significant challenge was the diversity of technologies across the 32 included studies. Interventions ranged from simple wearables like pedometers and accelerometers [31,44,47] to advanced systems like VR and exergaming [32,59], as well as telerehabilitation platforms [37,39]. This diversity made it challenging to categorize and analyze the technologies due to differences in technological complexity and functionality. The technologies were classified using the WHO DHI framework to streamline the analysis.

Another challenge was the inconsistency in study designs, outcomes, and reporting. Some studies were large-scale trials [39,42], while others were smaller pilot studies or case reports [36,38], making direct comparisons difficult. Moreover, few studies provided detailed long-term adherence data, limiting insights into the sustainability of interventions. Although some studies [31,44] reported high adherence to wearables and mHealth apps, the lack of longitudinal data restricted a comprehensive understanding of their long-term impact.

Access to technology and digital literacy were also significant barriers. Advanced interventions, such as VR and telerehabilitation [32,46], posed challenges for older patients or those with limited technological experience. Additionally, telerehabilitation platforms [37] required reliable internet access, which was not always available for patients in rural or low-resource settings, further contributing to disparities in access.

Moreover, there was a lack of personalized interventions. Most technologies followed a standardized approach, which may not have addressed the individual needs of cancer patients. Only a few studies [38] offered tailored interventions based on patient preferences or abilities, and this lack of customization may have impacted patient engagement and outcomes.

Finally, limited evidence exists on the cost-effectiveness of these technological interventions. While the studies demonstrated the feasibility and usability of wearables and mHealth apps [33,41], none provided a comprehensive cost analysis. This absence of cost-effectiveness data raises concerns about the scalability and equitable application of these interventions in broader clinical practice.

## 5. Conclusions

This scoping review mapped various technological resources used for physical rehabilitation in cancer patients undergoing chemotherapy, including wearables, mobile-based applications, telerehabilitation, web-based platforms, virtual reality, and exergames. These technologies may have the potential to improve physical function, manage symptoms, and enhance quality of life. However, despite the positive outcomes in the short term, their long-term effectiveness remains uncertain and requires further validation through clinical trials. The diversity of tools such as mHealth apps (e.g., the PINK! app) and wearables like Fitbit illustrates the broad range of options available for cancer rehabilitation, though personalization and sustained engagement are areas that need enhancement. A key challenge identified was patient adherence, particularly due to factors such as fatigue and low motivation, which are common in patients undergoing chemotherapy. However, technologies that provided real-time feedback, such as wearables and specific mHealth apps, showed promise in improving engagement with rehabilitation programs. Future research should prioritize the development of more personalized interventions tailored to patients’ individual needs and preferences, which can improve adherence and optimize rehabilitation outcomes.

Additionally, evaluating the cost-effectiveness and accessibility of these technologies is crucial, especially for underserved and low-resource populations. Overcoming barriers to access, including digital exclusion, is essential to ensure that these technological resources are equitably available to all cancer patients, thus maximizing their rehabilitation potential.

## Figures and Tables

**Table 2:** Technological resource types and categorization.

ID.	Technology Type	WHO DHI Classification	Functionality	Feasibility	Usability	Appropriateness/ Suitability	Objective	Key Findings
S01 [30]	Web-based application (EPT tool)	DHI 1.1—Client-targeted (therapy)	Exercise planning, mental health monitoring	High adherence, easy to use for home-based monitoring, no significant cost barriers.	Highly usable for remote use and adaptable for patients undergoing chemotherapy.	Well-suited for promoting physical activity and managing mental health in cancer patients.	Improved physical activity, mental health	Improved anxiety, depression, and walking distance.
S02 [31]	mHealth + Wearable device	DHI 1.2—Client-targeted (self-monitoring)	Exercise guidance, physical activity tracking	Good feasibility: mobile app and wearable devices are practical; adherence was 75%.	Mobile app and wearable are good self-monitoring tools suited for tracking fatigue and performance.	Appropriate for addressing fatigue and endurance, well-targeted for rehabilitation.	Improved strength, endurance, fatigue	Improved strength, endurance, and reduced fatigue.
S03 [32]	mHealth + VR	DHI 1.1—Client-targeted (therapy)	Group exercise, VR therapy	Requires technological familiarity, but feasible for patients comfortable with tech. Adherence varies.	Engaging, but requires more technological knowledge for use.	Suitable for comprehensive physical and psychological rehabilitation, including anxiety reduction.	Improved physical, psychological status	Improved BMI, anxiety, depression, and muscle mass.
S04 [33]	App-based coaching (PINK!)	DHI 1.4—Client-targeted (coaching)	Exercise, nutrition, mental health coaching	High adherence, practical for patients needing daily support.	Easy to use and provides regular support for psychological and physical health.	Highly suitable for fatigue reduction and mental health management.	Reduced distress, increase PA	Reduced fatigue, distress, and increased physical activity.
S05 [34]	Exergaming (Xbox Kinect)	DHI 1.1—Client-targeted (therapy)	Interactive gaming for physical activity	Feasible for younger patients or those familiar with gaming systems; cost may vary.	Interactive and engaging, but access to technology may limit use.	Well-suited for upper extremity rehabilitation.	Improve upper extremity functionality	Improved range of motion; no significant change in strength.
S06 [35]	Web-based exercise system (e-CuidateChemo)	DHI 1.1—Client-targeted (therapy)	Web-based exercise therapy	High adherence, feasible for remote intervention, accessible to patients with Internet access.	Easy to use for breast cancer patients at home; remote access ensures usability during chemotherapy.	Well-suited for mitigating chemotherapy-related physical deterioration.	Counters physical deterioration	Improved functional capacity and strength, no significant changes in body composition.
S07 [36]	Wearable (Polar V800 sports watch)	DHI 1.2—Client-targeted (self-monitoring)	High-intensity physical training tracking	Feasible for highly active patients but may not be suitable for all cancer patients due to high intensity.	Convenient for tracking fitness levels, but limited to patients capable of engaging in high-intensity activities.	Suitable for motivated, physically fit patients.	Tracks physical activity and fitness	Maintained fitness, completed marathons, improved physical fitness during cancer treatment.
S08 [37]	Telerehabilitation platform (eChez-Soi)	DHI 1.1—Client-targeted (remote care)	Remote exercise monitoring	High adherence, feasible for remote care with sensors, accessible for patients with internet access.	Highly usable for supervised remote rehabilitation, good adherence.	Very suitable for patients needing remote rehabilitation.	Improves functional capacity through remote exercise	High satisfaction, improved 6MWT, significant improvements in functional capacity, no adverse events.
S09 [38]	Wearable (Fitbit) + SMS	DHI 1.2—Client-targeted (self-monitoring)	Physical activity tracking, reminders	High adherence to Fitbit, practical due to automated SMS, though engagement with SMS was moderate.	Easy to use for tracking physical activity and providing reminders through SMS, though SMS engagement may vary.	Suitable for promoting physical activity during chemotherapy.	Promotes physical activity	Improved adherence to daily activity tracking, slight improvements in physical activity.
S10 [39]	Telerehabilitation platform	DHI 1.1—Client-targeted (remote care)	Remote exercise monitoring	Feasible for patients undergoing chemotherapy, high adherence, some technical issues reported but resolved.	Easy to use and practical for telerehabilitation programs with few technical problems.	Suitable for home-based rehabilitation for lung cancer patients.	Evaluates telerehabilitation for lung cancer patients	High adherence, reliable platform, minor technical issues, high patient satisfaction.
S11 [40]	eHealth app	DHI 1.1—Client-targeted (remote care)	Home-based exercise	High adherence (90%), feasible for multiple myeloma patients across various treatment stages.	Suitable for home-based virtual exercise, highly usable and adaptable for patient needs.	Well-suited for improving fitness and quality of life in multiple myeloma patients.	Improves fitness and quality of life	High adherence, improved physical fitness and quality of life, no adverse events.
S12 [41]	App-based coaching (PINK! Coach)	DHI 1.4—Client-targeted (coaching)	Coaching for PA, nutrition, stress management	Feasible for breast cancer patients undergoing chemotherapy, cost-effective for remote support, high adherence.	Practical and easy to use for maintaining physical activity and lifestyle changes.	Suitable for supporting weight management and physical activity in breast cancer patients.	Supports weight management during chemotherapy	Maintained or reduced BMI, increased physical activity, especially in antihormone therapy patients.
S13 [42]	mHealth (Go-EXCAP mobile app)	DHI 1.1—Client-targeted (remote care)	Remote-supervised exercise, monitoring	High adherence to wearables and remote monitoring, practical for improving fitness during chemotherapy.	Usable for patients requiring close monitoring during rehabilitation, accessible for home use.	Suitable for improving fitness and strength, well-targeted for remote interventions.	Improves fitness and strength	High adherence, improved fitness and strength through remote monitoring.
S14 [43]	Video conferencing (Meet app)	DHI 1.1—Client-targeted (remote care)	Supervised exercise, motivational strategies	Feasible for remote exercise programs, high engagement reported, accessible through standard video conferencing tools.	Highly usable for remote supervision, provides motivational support during exercise programs.	Suitable for colorectal cancer patients needing both physical and psychological support during chemotherapy.	Improves physical activity and psychological well-being	Improved physical activity and psychological well-being, high engagement, effective motivational strategies.
S15 [44]	Pedometer-based walking program	DHI 1.2—Client-targeted (self-monitoring)	Physical activity tracking	Feasible with high adherence, accessible for patients with limited physical activity experience, low-cost.	Easy to use pedometer-based programs, well-suited for tracking walking activity.	Suitable for improving physical activity and reducing fatigue, especially for patients undergoing chemotherapy.	Enhances physical activity, reduce fatigue	Improved physical activity, reduced fatigue.
S16 [45]	mHealth app (Smart Aftercare app)	DHI 1.2—Client-targeted (self-monitoring)	Physical activity, intensity tracking	High adherence, feasible for remote monitoring of exercise capacity	Easy to use for tracking physical activity intensity and providing real-time feedback for patients.	Well-suited for increasing physical activity intensity and tracking quality of life improvements.	Tracks PA, HRQoL	Increased physical activity and improved quality of life, especially exercise capacity improvements.
S17 [46]	Pedometer (ONWALK 100) + Telehealth platform	DHI 1.2—Client-targeted (self-monitoring)	Walking + weekly SMS reminders	Feasible, though fatigue poses challenges to adherence. Technologically accessible and affordable.	Usable for patients needing remote physical activity guidance, but fatigue reduced engagement	Suitable for encouraging physical activity during chemotherapy	Improves physical activity and QoL	Moderate improvements in physical activity and QoL; fatigue limited adherence to the intervention.
S18 [47]	Pedometer-based walking program (SILVA ex connect)	DHI 1.2—Client-targeted (self-monitoring)	Physical activity tracking via pedometer	High adherence, practical for tracking walking-based interventions	Usable for daily step tracking, accessible for home-based implementation	Suitable for maintaining physical activity and improving QoL during chemotherapy	Improves QoL, track daily steps	High adherence, improvements in daily steps and specific symptoms in breast and colorectal cancer patients.
S19 [48]	Pedometer-based walking program	DHI 1.2—Client-targeted (self-monitoring)	Physical activity tracking	Feasible with high adherence, accessible and low-cost, suitable for home use.	Simple and easy to use for tracking daily steps.	Suitable for enhancing physical activity and quality of life during chemotherapy.	Enhances physical activity, improves QoL	Significant improvements in physical activity and quality of life.
S20 [49]	Pedometer (Omron Walking Style Pro) + Multimodal intervention	DHI 1.2—Client-targeted (self-monitoring)	Multimodal rehabilitation (aerobic, strength, nutrition)	Feasible for leukemia patients during consolidation chemotherapy	Usable and practical for comprehensive rehabilitation, including physical activity and dietary support	Suitable for improving physical function, reducing fatigue, and supporting QoL during chemotherapy	Improves physical function, QoL, and reduce fatigue	Significant improvements in physical function, QoL, and reduction in fatigue and anxiety. Emotional well-being was also improved.
S21 [50]	Activity tracker-based PA program	DHI 1.2—Client-targeted (self-monitoring)	Physical activity tracking	Feasible, accessible with affordable technology, adherence varies based on patient motivation.	Easy to use for monitoring and increasing physical activity.	Well-suited for reducing sedentary behavior and improving activity levels during chemotherapy.	Monitors and increases physical activity	Increased physical activity and reduced sitting time.
S22 [51]	SenseWear accelerometer	DHI 1.2—Client-targeted (self-monitoring)	Physical activity, intensity tracking	Feasible for real-time intensity tracking, accessible for most patients, though technical requirements may challenge some users.	Easy to use for tracking activity intensity, providing useful feedback on physical activity levels.	Suitable for promoting moderate-to-vigorous physical activity and improving quality of life.	Tracks PA, provides feedback on intensity	Improved physical activity and quality of life through activity tracking and feedback.
S23 [52]	Walking program with pedometer	DHI 1.2—Client-targeted (self-monitoring)	Physical activity tracking	Feasible, with high adherence to walking-based programs. Low-cost and accessible, making it practical for patients undergoing chemotherapy.	Simple and easy to use, highly usable for tracking daily steps and improving physical activity.	Well-suited for tracking steps and monitoring fatigue during chemotherapy.	Tracks steps, monitors fatigue	Reduced fatigue and improved quality of life.
S24 [53]	activPAL accelerometer	DHI 1.2—Client-targeted (self-monitoring)	Sedentary behavior tracking	Feasible with good adherence, accessible, though adherence may vary for patients with advanced cancers.	Easy to use for tracking sedentary behavior and motivating patients to increase movement.	Well-suited for advanced cancer patients needing to reduce sedentary behavior.	Tracks sedentary behavior	Improved quality of life and reduced sedentary behavior in advanced cancer patients.
S25 [54]	Misfit Shine activity tracker (eHealth interventions + wearable devices)	DHI 1.2—Client-targeted (self-monitoring)	Physical activity tracking	Feasible with good adherence, affordable, and accessible for daily use; effective for tracking physical activity.	Easy to use, highly usable for monitoring daily activity.	Suitable for improving physical activity and quality of life during chemotherapy or cancer treatment.	Monitors daily physical activity	Improved physical activity and quality of life with daily activity tracking.
S26 [55]	SenseWear Armband	DHI 1.2—Client-targeted (self-monitoring)	Moderate-to-vigorous physical activity tracking	Feasible with high adherence, accessible for tracking MVPA, affordable, and practical for cancer rehabilitation.	Easy to use for real-time physical activity monitoring, providing motivational feedback.	Well-suited for promoting MVPA and reducing cancer-related fatigue.	Tracks MVPA	Reduced fatigue, increased moderate-to-vigorous physical activity.
S27 [56]	Pedometer-based walking program	DHI 1.2—Client-targeted (self-monitoring)	Physical activity promotion	Feasible with high adherence, low-cost, and practical for increasing physical activity through walking.	Easy to use for promoting adherence to walking programs during cancer treatment.	Suitable for encouraging physical activity and adherence to rehabilitation programs during chemotherapy.	Encourages adherence to PA program	High adherence, but no significant difference in physical activity between groups.
S28 [57]	Walking-based outdoor activity (Pedometer)	DHI 1.2—Client-targeted (self-monitoring)	Outdoor activity tracking	Feasible with good adherence, practical for promoting outdoor activity, requires patient motivation.	Usable and simple to implement for increasing outdoor physical activity.	Well-suited for improving quality of life in patients with advanced cancer.	Improves QoL	Increased outdoor activity, improved quality of life in patients with NSCLC and pancreatic cancer.
S29 [58]	ActiGraph accelerometer	DHI 1.2—Client-targeted (self-monitoring)	Activity tracking for personalized PA	Feasible with high adherence, practical for personalized physical activity interventions.	Easy to use for personalized physical activity tracking.	Suitable for increasing MVPA through personalized interventions in cancer patients.	Monitors activity levels	High adherence, increased MVPA through personalized PA intervention.
S30 [59]	Kinect-based rehabilitation	DHI 1.1—Client-targeted (therapy)	Interactive rehabilitation, fatigue reduction	Feasible for patients with access to gaming systems, high engagement, though some patients may find cost or accessibility a barrier.	Highly usable for interactive rehabilitation, engaging for patients.	Well-suited for reducing fatigue and improving quality of life during cancer treatment.	Improves QoL	Reduced fatigue, improved quality of life through interactive rehabilitation.
S31 [60]	Kinect-based exercise program	DHI 1.1—Client-targeted (therapy)	Shoulder mobility, exercise therapy	Feasible for shoulder mobility improvement, though cost and access to gaming technology may limit feasibility for some patients.	Usable for patients needing rehabilitation of shoulder mobility.	Suitable for improving shoulder mobility, though not as effective for muscle strength improvement.	Improves shoulder mobility, reduces disability	Reduced shoulder disability, no significant changes in muscle strength.
S32 [61]	Wii Fit-based exercise program (exergaming and VR)	DHI 1.1—Client-targeted (therapy)	Physical performance, psychological support	Feasible for patients with access to gaming systems, moderate adherence, practical for psychological support and physical performance maintenance.	Usable and engaging, though adherence varies based on patient condition and technological access.	Suitable for maintaining physical performance and supporting mental health during chemotherapy.	Maintains physical performance, improves mental health	Maintained physical performance, improved anxiety and depression in hematologic malignancy patients.

WHO DHI: World Health Organization Digital Health Intervention; PA: physical activity; MVPA: moderate-to-vigorous physical activity; QoL: quality of life; 6MWT: Six-Minute Walk Test; BMI: Body Mass Index; HRQoL: health-related quality of life; NSCLC: non-small cell lung cancer.

## Appendix A

**Table A1:** Research strategies according to the database.

Database	Research Strategy
Medline	(((MH “Neoplasms”) OR (“Cancer patients”) OR (MH “Cancer Survivors”) OR (MH “Chemotherapy, Adjuvant”) OR (MH “Antineoplastic Agents”) OR (“Chemotherapy”)) AND ((MH “Technology”) OR (MH “Health Resources”) OR (MH “Digital Technology”) OR (MH “Biomedical Technology”) OR (MH “Digital Health”) OR (MH “Wearable Electronic Devices”) OR (“technological resources”)) AND ((MH “Rehabilitation”) OR (MH “Physical Therapists”) OR (MH “Rehabilitation Nursing”) OR (MH “Physical and Rehabilitation Medicine”) OR (“physical rehabilitation”) OR (MH “Physical Education and Training”) OR (MH “Exercise”) OR (MH “Telerehabilitation”) OR (MH “Exercise Therapy”) OR (“Exercise”)))
CINAHL	(((MM “Cancer Patients”) OR (MH “Antineoplastic Agents”) OR (MH “Chemotherapy, Cancer”) OR (MH “Chemotherapy, Adjuvant”) OR (“Cancer patients”) OR (“Cancer Survivors”) OR (“Chemotherapy”)) AND ((MH “Health Resource Utilization”) OR (MH “Assistive Technology Services”) OR (MH “Telerehabilitation”) OR (MH “Digital Technology”) OR (“Technology”) OR (“Health Resources”) OR (“Digital Technology”) OR (“Biomedical Technology”) OR (“Digital Health”) OR (“Wearable Electronic Devices”) OR (“technological resources”) OR (“eHealth”)) AND ((MH “Rehabilitation”) OR (MH “Rehabilitation, Cancer”) OR (MH “Therapeutic Exercise”) OR (MH “Aerobic Exercises”) OR (MH “Muscle Strengthening”) OR (MH “Physical and Rehabilitation Medicine”) OR (MM “Exercise Therapy”) OR (“Physical Therapists”) OR (“Rehabilitation Nursing”) OR (“Physical and Rehabilitation Medicine”) OR (“Physical Rehabilitation”) OR (“Physical Education and Training”) OR (“Exercise”) OR (“Telerehabilitation”) OR (“Exercise Therapy”)))
Sport Discus	(((DE “CANCER chemotherapy”) OR (DE “CANCER patients”) OR (“Cancer patients”) OR (“Chemotherapy”)) AND ((DE “SPORTS & technology”) OR (“Digital Health”) OR (“Technological Resources”) OR (“Technology”) OR (“Health Resources”) OR (“Digital Technology”) OR (“Biomedical Technology”) OR (“Wearable Electronic Devices”)) AND ((DE “PHYSICAL therapy”) OR (DE “EXERCISE”) OR (DE “EXERCISE therapy”) OR (“Rehabilitation”) OR (“Physical Therapists”) OR (“Rehabilitation Nursing”) OR (“Physical and Rehabilitation Medicine”) OR (“physical rehabilitation”) OR (“Physical Education and Training”) OR (“Exercise”) OR (“Telerehabilitation”) OR (“Exercise Therapy”)))
Scopus	(TITLE-ABS-KEY ((“Chemotherapy Cancer”) OR (“Chemotherapy”) OR (“Antineoplastic Agents”)) AND TITLE-ABS-KEY ((“Technology”) OR (“Health Resources”) OR (“Digital Technology”) OR (“Biomedical Technology”) OR (“Digital Health”) OR (“Wearable Electronic Devices”) OR (“technological resources”)) AND TITLE-ABS-KEY ((“Exercise”) OR (“Exercise Therapy”) OR (“Rehabilitation”) OR (“physical rehabilitation”) OR (“Rehabilitation Cancer”)))
Cochrane	(((MH “Neoplasms”) OR (“Cancer patients”) OR (MH “Cancer Survivors”) OR (MH “Chemotherapy, Adjuvant”) OR (MH “Antineoplastic Agents”) OR (“Chemotherapy”)) AND ((MH “Technology”) OR (MH “Health Resources”) OR (MH “Digital Technology”) OR (MH “Biomedical Technology”) OR (MH “Digital Health”) OR (MH “Wearable Electronic Devices”) OR (“technological resources”)) AND ((MH “Rehabilitation”) OR (MH “Physical Therapists”) OR (MH “Rehabilitation Nursing”) OR (MH “Physical and Rehabilitation Medicine”) OR (“physical rehabilitation”) OR (MH “Physical Education and Training”) OR (MH “Exercise”) OR (MH “Telerehabilitation”) OR (MH “Exercise Therapy”) OR (“Exercise”)))
